# Predictability of Nonremitting Depression After First 2 Weeks of Antidepressant Treatment: A VAST‐D Trial Report

**DOI:** 10.1176/appi.prcp.20190003

**Published:** 2019-10-03

**Authors:** Paul B. Hicks, Varadan Sevilimedu, Gary R. Johnson, Ilanit Tal, Peijun Chen, Lori L. Davis, Julia E. Vertrees, Somaia Mohamed, Sidney Zisook

**Affiliations:** ^1^ Department of Psychiatry Baylor Scott & White Health; ^2^ Texas A&M College of Medicine Temple Texas; ^3^ Yale University School of Public Health New Haven Connecticut; ^4^ Cooperative Studies Program Coordinating Center Veterans Affairs (VA) Connecticut Healthcare System West Haven; ^5^ VA San Diego Healthcare System San Diego; ^6^ Louis Stokes Cleveland VA Medical Center Clevelend; ^7^ Tuscaloosa VA Medical Center Tuscaloosa Alabama; ^8^ University of Alabama School of Medicine Birmingham; ^9^ Cooperative Studies Program Clinical Research Pharmacy Coordinating Center Albuquerque New Mexico; ^10^ VA New England Mental Illness Research, Education, and Clinical Center VA Connecticut Healthcare System West Haven; ^11^ Department of Psychiatry University of California San Diego

**Keywords:** Early improvement, Antidepressant response, Negative predictive value, Treatment‐resistant depression

## Abstract

**Objective:**

In this secondary analysis of data from the Veterans Affairs Augmentation and Switching Treatments for Improving Depression Outcomes (VAST‐D) study, the authors sought to determine the effectiveness of early improvement (or lack thereof) for predicting remission from depression with antidepressant therapy.

**Methods:**

This study used data from the VAST‐D study, a multisite, randomized, single‐blind trial with parallel assignment to one of three medication interventions for 1,522 veterans whose major depressive disorder was unresponsive to at least one course of antidepressant treatment meeting minimal standards for dosage and duration. The authors calculated the positive predictive value (PPV) and negative predictive value (NPV) of early improvement on remission, response, or greater than minimal improvement from depression for various degrees of improvement (10%–50%) on the Quick Inventory of Depressive Symptomatology–Clinician Rated (QIDS‐C) at 1, 2, 4, and 6 weeks.

**Results:**

The end of week 2 of treatment was identified as the best time to evaluate early improvement. The presence of a ≥20% drop from the baseline QIDS‐C score by the end of week 2 resulted in a PPV for remission of 38% and an NPV of 93% by week 12. Extending the observational window to week 6 minimally improved NPV (97%). This association did not differ across treatment groups.

**Conclusions:**

A lack of early improvement at the end of week 2 of antidepressant therapy can be used to inform clinical decisions on the likelihood of nonremission of depression during the subsequent 10 weeks, even when dosage optimization is incomplete.

**HIGHLIGHTS**

- The optimal time for evaluating early improvement from an antidepressant medication regimen is at the end of week 2.
- A lack of early improvement at the end of week 2 of antidepressant therapy can be used to inform clinical decisions on the likelihood of nonremission of depression with that therapy during the subsequent 10 weeks, even when dosage optimization is incomplete.
- The same factors that influence early improvement also determine whether a patient will show a false negative outcome (i.e., achieve remission by the end of week 12 despite no early improvement): lower baseline Quick Inventory of Depressive Symptomatology–Clinician Rated score, fewer adverse childhood experiences, lower baseline anxiety, lower suicidal ideation, and higher baseline quality of life score.
- The utility of using lack of early improvement to predict lack of remission in antidepressant therapy did not depend on treatment allocation.

Major depressive disorder is a significant health concern not only because it is one of the most prevalent psychiatric disorders (1), but because it accounts for the greatest number of disability‐adjusted life years among psychiatric disorders (2). Proper management is therefore critical. There is consensus about which drugs to choose at the initiation of antidepressant medication therapy (e.g., a selective serotonin reuptake inhibitor [SSRI]) and what the target dosages of these drugs should be (3). It is also generally agreed that the optimal endpoint should be remission of symptoms and that it is prudent for clinicians to adjust the medication therapy until remission is achieved (4, 5, 6). However, when antidepressant medication therapy does not result in the expected improvement, the decision‐making process becomes complicated. For example, if remission is not achieved, should the clinician accept a lower level of improvement? Also, when should the first decision‐point occur? Knowing when to alter the medication treatment and knowing the probability of achieving greater improvement at each decision point could save weeks to months of unnecessary suffering and minimize the adverse consequences of ineffectively treated depression.

In the management of depression of patients who do not adequately respond to initial therapy, it is critical to determine when a patient will need to proceed to a next‐step medication. In a meta‐analysis covering 17 studies and 14,779 patients, the role of early improvement (i.e., a ≥20% drop from baseline depression severity score on either the Hamilton Depression Rating Scale or the Montgomery‐Asberg Depression Rating Scale at the end of 2 weeks of medication therapy) was assessed (7). Nearly two‐thirds (63%) of patients treated with an antidepressant showed early improvement, whereas only 47% of patients treated with placebo did. The use of early improvement accurately predicted those patients who would ultimately achieve remission by 8–12 weeks in 42% of the patients (positive predictive value [PPV]); more importantly, the absence of a ≥20% early improvement predicted the lack of ultimate remission for 90% of the patients (negative predictive value [NPV]). Early improvers were 8.4 times more likely to be identified as a later responder to the medication and 6.4 times more likely to achieve remission than a patient who showed no early improvement. Other meta‐analyses, evaluating data on fewer participants, have also provided evidence supporting early improvement as a predictor of ultimate remission (8, 9, 10, 11). In these studies, lack of early improvement has been the most reliable predictor of nonremission. In addition, in one meta‐analysis, a slightly higher NPV (94%) was noted when the early improvement observation period was extended to 4 weeks (10).

The Veterans Affairs Augmentation and Switching Treatments for Improving Depression Outcomes (VAST‐D) study is the largest next‐step trial for individuals who did not adequately respond to an initial antidepressant (4, 12, 13). Our goal in this secondary analysis of the VAST‐D data was to explore the effectiveness of using early improvement (i.e., a drop from the baseline depression severity score as measured by the Quick Inventory of Depressive Symptomatology–Clinician Rated [QIDS‐C] within the first few weeks of antidepressant treatment) to predict remission, response, or greater than minimal improvement during the acute phase of the trial (the first 12 weeks of treatment).

## Methods

### Compliance

The U.S. Department of Veterans Affairs (VA) Office of Research and Development and the Central Institutional Review Board (CIRB) approved the VAST‐D study. A certificate of confidentiality was obtained for the study from the National Institutes of Health. The CIRB conducted annual continuing reviews, and a data monitoring committee (DMC) reviewed the study biannually. Adverse events were reviewed by both the CIRB and DMC throughout the study. All participants provided written informed consent and privacy authorization after receiving full explanation of the study procedures.

### Study Design

VAST‐D was a multisite (see the online supplement for a list of participating sites), randomized, single‐blind, parallel‐assignment next‐step trial of veterans whose major depressive disorder was suboptimally responsive to at least one course of antidepressant treatment with an SSRI, serotonin and norepinephrine reuptake inhibitor, or mirtazapine that met or exceeded minimal standards for dosage and duration of treatment. Suboptimal response was defined as a score of ≥16 (indicating severe depression) on the QIDS‐C questionnaire after at least 6 weeks of treatment or a score of ≥11 (indicating moderate depression) after at least 8 weeks of treatment, with the three most recent weeks at a stable, “optimal” dosage (4, 12, 13).

A full description of the overall design of the VAST‐D study (including the Consolidated Standards of Reporting Trials [CONSORT]) statement and flow diagram) has been published previously (4, 12, 13).

### Participants

Participants were 1,522 Veterans Health Administration (VHA) patients, 18 years or older and diagnosed as having major depressive disorder, who were referred by their VHA clinicians. Study clinicians confirmed the diagnosis prior to study enrollment. Research staff further established diagnostic eligibility using criteria from the *DSM‐IV‐TR*. Potential participants who were pregnant; breastfeeding; currently using contraindicated medications, including either study drug; or had a clear history of nonresponse or intolerance to bupropion‐SR or aripiprazole, were excluded from the study. Participants who had a primary diagnosis of bipolar, psychotic, obsessive‐compulsive, dementia, or eating disorders; had general medical conditions contraindicating the use of bupropion‐SR or aripiprazole; had serious, unstable medical conditions requiring acute treatment; met criteria for substance dependence requiring inpatient detoxification; or were considered at high risk for suicide and in need of acute treatment were also excluded.

### Interventions

This report addresses the acute phase (first 12 weeks of treatment) of the VAST‐D study, in which 1,522 veterans with nonpsychotic major depressive disorders were randomized to one of three treatment groups: augmentation with bupropion‐SR (Aug‐BUP), augmentation with aripiprazole (Aug‐ARI), or switch to another antidepressant (i.e., bupropion‐SR [Switch‐BUP]) (4, 12, 13). For the treatment groups receiving them, the dosage of index antidepressants remained relatively constant throughout the trial. Treatments included titration (cross‐titration for the Switch‐BUP group) from standard starting dosages of 150 mg bupropion‐SR with titration up to 400 mg daily or 2 mg aripiprazole with titration up 15 mg daily, until depressive symptoms remitted or side effects were intolerable. Dosage adjustments were guided by participant responses on the Patient Health Questionnaire (14) and a Frequency, Intensity, and Burden of Side Effects Rating (15) obtained at each visit. Treatment visits occurred at baseline and at the end of weeks 1, 2, 4, 6, 8, 10, and 12.

### Baseline Assessments

Baseline measures in this analysis included age, marital status, education, employment status, race‐ethnicity, number of lifetime episodes of major depressive disorder, duration of the current episode, number of past medication trials with antidepressants, presence of a substance or alcohol abuse diagnosis (Mini‐International Neuropsychiatric Interview score) (16), severity of childhood adverse experiences (Adverse Childhood Experiences Survey score ) (17), severity of grief (Complicated Grief Questionnaire score) (18), severity of suicidal ideation (Columbia‐Suicide Severity Rating Scale [C‐SSRS] score) (19), severity of anxiety (Beck Anxiety Inventory score) (20), presence of mixed features as measured by a self‐rated 9‐item mixed features scale based on the *DSM‐5*, severity of health impairment as measured by the Cumulative Illness Rating Scale (CIRS) (21), general life satisfaction as measured by the Quality of Life Enjoyment and Satisfaction Questionnaire–Short Form (Q‐LES‐Q‐SF) (22), QIDS‐C score (23), and duration of the index treatment trial (in months).

### Primary Outcome Measure

The primary outcome measure, the QIDS‐C score, was collected by an independent evaluator who was blind to the patients’ treatment assignments at baseline and at each visit following randomization. Standard definitions of “response” (≥50% decrease from baseline QIDS‐C score at the end of week 12), and “remission” (QIDS‐C scores ≤5 on two consecutive evaluations anytime during the 12‐week acute phase) were used. In addition, “greater than minimal improvement” was defined as a >30% decrease from baseline QIDS‐C score at the end of week 12. Except in exploratory analyses, early improvement was defined as a ≥20% drop from baseline QIDS‐C score by the end of week 2.

### Statistical Analysis

We conducted the statistical analysis by using observed cases. We calculated the PPV and NPV of early improvement on remission. To calculate PPV and NPV, we categorized participant outcomes as true positive (TP), false positive (FP), true negative (TN), and false negative (FN). A TP outcome was defined as having a ≥20% drop from baseline QIDS‐C score by the end of week 2 (early improvement) and achieving remission by the end of week 12. A FP outcome was defined as showing early improvement but not achieving remission by the end of week 12. A TN outcome was one in which the participant did not demonstrate early improvement and did not achieve remission by the end of week 12. A FN outcome was one in which the participant did not show early improvement but achieved remission by the end of week 12. PPV and NPV were calculated as PPV=TP/(TP+FP) and NPV=TN/(TN+FN). We calculated sensitivity as the ratio of true positive outcomes to the total number of patients achieving remission (sensitivity =TP/[TP+FN]) and specificity as the ratio of true negative outcomes to the total number of patients not achieving remission (specificity=TN/[TN+FP]). The relative likelihood of remission, response, and greater than minimal improvement between those displaying early improvement and those who did not was calculated as the unadjusted odds ratios from 2×2 frequency tables.

To identify the optimal drop in baseline QIDS‐C score and the observational window to achieve the best PPV and NPV values, we calculated the PPVs and NPVs for multiple percentage drops (10%, 20%, 30%, 40%, and 50%) and at various observational windows (weeks 1, 2, 4, and 6).

We identified baseline characteristics associated with early responders and participants exhibiting false negative outcomes by using chi‐square tests for categorical variables and Wilcoxon rank sum tests for continuous variables. We calculated effect sizes (Cohen's d) as the difference of the means divided by the pooled standard deviation. We conducted a chi‐square analysis to compare withdrawal rates between early improvers and those who did not have early improvement. We used chi‐square analysis to perform area‐under‐the‐curve comparisons of receiver operating curves to determine the generalizability of using early improvement to predict remission.

## Results

Sixty‐two percent of the sample showed a ≥20% drop from the baseline QIDS‐C score by the end of week 2 (early improvement). Table 1 shows that early improvement resulted in a PPV for remission of 38% and an NPV for remission of 93%. The odds of achieving remission, response, and greater than minimal improvement was higher among individuals who exhibit early improvement (odds ratio [OR]=7.7, 95% confidence interval [CI]=5.4–11.1; OR 3.5, 95% CI=2.7–4.6; and OR 3.6, 95% CI=2.7–4.9, respectively). The corresponding sensitivity and specificity for remission were 91% (95% CI=87.6–93.5) and 44% (95% CI=40.6–46.7), respectively.

**Table 1:** Calculation of the positive predictive value (PPV) and negative predictive value (NPV) for a ≥20% drop in QIDS‐C score from baseline to the end of week 2 (early improvement) among 1,522 veterans with depression^a^

	**Early improvement**				**No early improvement**					
**Patient status at end of week 12**	**TP** ^b^ **(N)**	**FP** ^c^ **(N)**	**PPV (%)**	**95% CI**	**FN** ^d^ **(N)**	**TN** ^e^ **(N)**	**NPV (%)**	**95% CI**	**OR** ^f^	**95% CI**
Remission^g^	359	581	38.2	35.0–41.4	36	450	92.6	90.3–94.9	7.7	5.4–11.1
Response^h^	527	242	68.5	66.5–70.5	131	212	61.8	57.4–66.0	3.5	2.7–4.6
GTMI^i^	658	111	85.6	83.7–87.2	213	130	37.9	34.1–41.8	3.6	2.7–4.9

a QIDS‐C, Quick Inventory of Depressive Symptomatology–Clinician Rated. Possible scores range from 0 to 27, with higher scores indicating greater severity of depression.

b TP, true positives (participants who exhibited a ≥20% drop in QIDS‐C score by the end of week 2 [early improvement] and who achieved remission by the end of week 12).

c FP, false positives (participants who showed early improvement but did not achieve remission by the end of week 12).

d FN, false negatives (participants who did not show early improvement but achieved remission by the end of week 12).

e TN, true negatives (participants who did not demonstrate early improvement and did not achieve remission by the end of week 12.

f OR, odds ratio=(true positives)×(true negatives)/(false positives)×(false negatives).

g Remission was defined as QIDS‐C scores ≤5 on two consecutive evaluations anytime during the 12‐week acute phase (analysis included all participants with a week 2 assessment [N=1,426]).

h Response was defined as a ≥50% drop from baseline QIDS‐C score at the end of week 12 (analysis included only participants with a week 2 assessment who completed follow‐up to week 12 [N=1,112]).

i GTMI, greater than minimal improvement, was defined as a >30% drop from baseline QIDS‐C score at the end of week 12 (analysis included only participants with a week 2 assessment who completed follow‐up to week 12 [N=1,112]).

At baseline, early improvers were more likely to have been allocated to receive Aug‐ARI, have a greater number of lifetime episodes of depression, have less severe suicidal ideation, less anxiety, and higher quality of life (Table 2), although the effect sizes for these associations were small (Cohen's d=0.12–0.25). The highest level of education attained, marital status, employment status, presence of substance abuse, severity of grief, baseline QIDS‐C score, age at enrollment, number of lifetime antidepressant trials, severity of childhood adverse experiences, presence of mixed features as measured by a self‐rated 9‐item mixed features scale based on the *DSM‐5*, severity of health impairment (as measured by the CIRS), and duration of index treatment trial did not influence whether early improvement was present. Patients who did not have early improvement but achieved remission during the trial (i.e., had a false negative outcome) were more likely to have a lower baseline QIDS‐C score, fewer adverse childhood experiences, lower baseline Beck Anxiety Inventory score, lower C‐SSRS score, and a higher baseline quality of life (Q‐LES‐Q‐SF) score (Table 3).

**Table 2:** Characteristics of early improvers and early nonimprovers among 1,522 veterans with depression^a^

	Early improvers(N=940)		**Early nonimprovers(N=486)**			
**Characteristic**	**N**	**%**	**N**	**%**	**p**	**Cohen's d**
Treatment allocation					.008	NA
Switch‐BUP	293	31.2	180	37.0		
Aug‐BUP	309	32.9	169	34.8		
Aug‐ARI	338	36.0	137	28.2		
Education					.578	NA
Some college	366	38.9	187	38.5		
High school or less	256	27.2	145	29.8		
Associate's degree	131	13.9	57	11.7		
Bachelor's or higher	187	19.9	97	20.0		
Marital status					.468	NA
Married/cohabitating	428	45.5	219	45.1		
Divorced/separated	345	36.7	186	38.3		
Never married	129	13.7	69	14.2		
Widowed	38	4.0	12	2.5		
Employment status					.308	NA
Employed	250	26.6	113	23.3		
Retired	295	31.4	152	31.3		
Unemployed	392	41.7	220	45.3		
Substance or alcohol abuse					.313	NA
Yes	126	13.4	56	11.5		
No	814	86.6	430	88.5		
CGQ^b^					.190	NA
≤3	398	42.3	188	38.7		
>3	542	57.7	298	61.3		
QIDS‐C^c^ (M±SD)	16.7±3.22		16.6±3.33		.387	NA
Age (M±SD years)	54.1±12.42		55.0±11.58		.409	NA
Lifetime episodes of depression (M±SD)	2.64±1.35		2.45±1.37		.012	.14
Lifetime antidepressant trials (M±SD)	2.33±1.72		2.42±1.66		.084	NA
ACES^d^ (M±SD)	3.15±2.51		3.17±2.60		.98	
C‐SSRS^e^ (M±SD)	.75±1.21		.90±1.30		.016	.12
BAI^f^ (M±SD)	.86±.52		.99±.54		<.0001	.25
*DSM‐5* mixed features^g^ (M±SD)	11.6±2.59		11.6±2.56		.78	NA
CIRS^h^ (M±SD)	1.83±.38		1.80±.35		.11	NA
Q‐LES‐Q‐SF^i^ (M±SD)	42.1±14.3		38.6±14.1		<.0001	.25

a Early improvers, participants with ≥20% drop from baseline Quick Inventory of Depressive Symptomatology–Clinician Rated (QIDS‐C) score by the end of week 2. Frequencies and percents are used for categorical variables and means and standard deviations for continuous variables.

b CGQ, Complicated Grief Questionnaire. Possible scores range from 0 to 10, with higher scores indicating greater complicated grief.

c QIDS‐C**_,_** Quick Inventory of Depressive Symptomatology–Clinician Rated. Possible scores range from 0 to 27, with higher scores indicating greater severity of depression.

d ACES, Adverse Childhood Experiences Survey. Possible scores range from 0 to 10, with higher scores indicating greater childhood adversity and greater risk of psychological or health problems.

e C‐SSRS, Columbia Suicide Severity Rating Scale‐Suicidal Ideation. Possible scores range from 0 to 5, with higher scores indicating greater suicidal ideation or intent.

f BAI, Beck Anxiety Inventory. Possible scores range from 0 to 3 (average rating of each of the 21 items), with higher scores indicating greater anxiety.

g *DSM‐5* mixed features, presence of mixed features by a self‐rated 9‐item mixed features scale based on the *DSM‐5*. Possible scores range from 9 to 27, with higher scores indicating more hypomanic or manic symptoms.

h CIRS, Cumulative Illness Rating Scale Comorbidity Index. Possible scores range from 0 to 4, with higher scores indicating greater severity of co‐occurring medical conditions.

i Q‐LES‐Q‐SF, Quality of Life Enjoyment and Satisfaction Questionnaire–Short Form. Possible scores range from 0% to 100% of the maximum scale score of 70, with higher scores indicating greater life satisfaction and enjoyment.

**Table 3:** Baseline measures influencing achievement of remission by week 12 despite no early improvement (false negative outcome)

	**False negative (N=36)**		**True negative (N=450)**			
**Baseline Measure**	**N**	**%**	**N**	**%**	**p**	**Cohen's d**
Treatment allocation					0.7	NA
Switch‐BUP	11	30.6	169	37.6		
Aug‐BUP	14	38.9	155	34.4		
Aug‐ARI	11	30.6	126	28.0		
Education					0.53	NA
Some college	14	38.9	173	38.4		
High school or less	13	36.1	132	29.3		
Associate's degree	5	13.9	52	11.6		
Bachelor's degree or higher	4	11.1	93	20.7		
Marital status					0.45	NA
Married/cohabitating	13	36.1	206	45.8		
Divorced/separated	17	47.2	169	37.6		
Never married	6	16.7	63	14.0		
Widowed	0	0.0	12	2.7		
Employment status					0.5	NA
Employed	10	27.8	103	22.9		
Retired	13	36.1	139	30.9		
Unemployed	13	36.1	207	46.1		
Substance or alcohol abuse					0.78	NA
Yes	3	8.3	53	11.8		
No	33	91.7	397	88.2		
CGQ^a^					0.7	NA
≤3	15	41.7	173	38.4		
>3	21	58.3	277	61.6		
QIDS‐C^b^ (M±SD)	14.0±3.1		16.7±3.3		<0.001	0.84
Age (M±SD years)	53.7±13.3		55.0±11.4		0.56	NA
Lifetime episodes of depression (M±SD)	2.6±1.2		2.4±1.4		0.27	NA
Lifetime antidepressant trials (M±SD)	2.6±1.9		2.4±1.6		0.72	
ACES^c^ (M±SD)	2.3±2.2		3.2±2.6		0.04	0.37
C‐SSRS^d^ (M±SD)	0.52±1.1		0.93±1.3		0.05	0.34
BAI^e^ (M±SD)	0.71±0.5		1.01±0.5		0.001	0.61
*DSM‐5* mixed features^f^ (M±SD)	11.0±2.1		11.7±2.6		0.20	NA
CIRS^g^ (M±SD)	1.7±0.39		1.80±0.34		0.42	NA
Q‐LES‐Q‐SF^h^ (M±SD)	46.2±13.6		38.0±13.9		<0.001	0.59

a CGQ, Complicated Grief Questionnaire. Possible scores range from 0 to 10, with higher scores indicating greater complicated grief.

b QIDS‐C**_,_** Quick Inventory of Depressive Symptomatology–Clinician Rated. Possible scores range from 0 to 27, with higher scores indicating greater severity of depression.

c ACES, Adverse Childhood Experiences Survey. Possible scores range from 0 to 10, with higher scores indicating greater childhood adversity and greater risk of psychological or health problems.

d C‐SSRS, Columbia Suicide Severity Rating Scale‐Suicidal Ideation. Possible scores range from 0 to 5, with higher scores indicating greater suicidal ideation or intent.

e BAI, Beck Anxiety Inventory. Possible scores range from 0 to 3 (average rating of each of the 21 items), with higher scores indicating greater anxiety.

f *DSM‐5* mixed features, presence of mixed features by a self‐rated 9‐item mixed features scale based on the *DSM‐5*. Possible scores range from 9 to 27, with higher scores indicating more hypomanic or manic symptoms.

g CIRS, Cumulative Illness Rating Scale Comorbidity Index. Possible scores range from 0 to 4, with higher scores indicating greater severity of co‐occurring medical conditions.

h Q‐LES‐Q‐SF, Quality of Life Enjoyment and Satisfaction Questionnaire‐Short Form. Possible scores range from 0% to 100% of the maximum scale score of 70, with higher scores indicating greater life satisfaction and enjoyment.

Of the 940 participants who met the criterion for early improvement, 143 were withdrawn from the study (15%) for various reasons that have been described previously (12). Of the 582 participants who did not meet the criterion for early improvement, 171 (29%) were withdrawn from the study during the acute phase of treatment. These rates were significantly different according to a chi‐square analysis (χ^2^=23.53, df=1, p<0.001).

**Figure 1:**
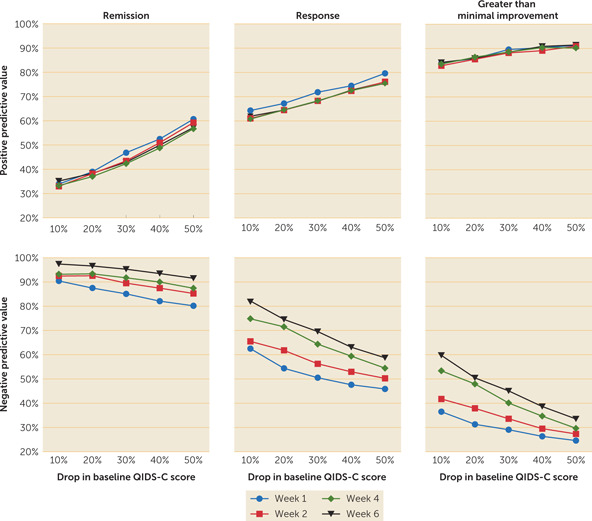
Positive predictive values and negative predictive values based on percentage drop from baseline QIDS‐C score over various observational periods for remission, response, and greater than minimal improvement^a^

**Figure 2:**
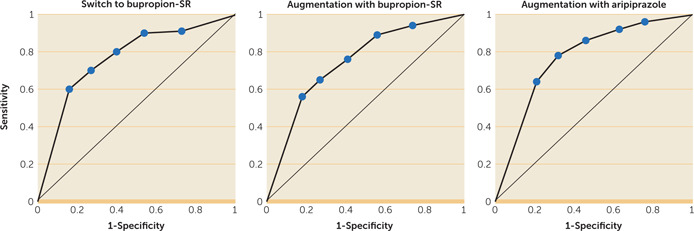
Influence of treatment group on predictive value of early improvement at week 2^a^

The PPV for remission was mostly influenced by the magnitude of the percentage drop in QIDS‐C score from baseline, with no obvious benefit provided by the duration of the observation window (Figure 1). The NPV, in contrast to the PPV, was influenced to some extent by the duration of the observation window. The NPV for greater than minimal improvement was low to moderate at all observation periods evaluated. While the use of at least a 20% drop from the baseline QIDS‐C score by the end of week 2 may not provide the strongest NPV for remission, NPV improved only 4% (from 93% to 97%) when we extended the observation period to 6 weeks. Receiver operating curves for the ability of early improvement at week 2 to predict remission as a function of treatment allocation are presented in Figure 2. Area‐under‐the‐curve comparisons did not support an influence of treatment on the predictive ability of early improvement.

a

a

The average prescribed dosages of bupropion at the end of week 2 were 237 mg and 221 mg for the Switch‐BUP and Aug‐BUP groups, respectively. The average dosage of aripiprazole at the end of week 2 was 3 mg (a full description of average dosages by time observation point is provided in a table in the online supplement).

## Discussion and Conclusions

For any antidepressant medication trial, it is important to identify as early as possible whether the patient is likely to achieve remission with the current treatment regimen. In this analysis of the VAST‐D study, which consisted of participants who were inadequately responsive to an initial antidepressant trial, we demonstrated that 62% exhibited a ≥20% drop from the baseline QIDS‐C score by the end of week 2 and that this early improvement (or lack of improvement) had a PPV of 38% and an NPV of 93% for prediction of remission by the end of week 12. In addition, our data show that those who reached the 20% threshold of early improvement by week 2 were more likely by the end of week 12 to achieve greater than minimal improvement or response, compared with patients who did not show this level of early improvement. In a smaller study of participants who had not responded to an initial antidepressant trial, venlafaxine was the only antidepressant studied (10). The data from that study suggested a greater benefit from assessing improvement at the end of week 4 instead of week 2, although the magnitude of the NPV and the pattern of the NPV acting as a better predictor than the PPV were similar. In that study, predictive values were evaluated only at weeks 2 and 4 for >20% or >30% drops from the baseline depression score. In the present study, we systematically studied multiple time observation windows and percentage drops from the baseline depression score. We also allowed dosage adjustment as early as the end of week 1. This difference may have contributed to the higher NPV values. Early improvement was also found to be useful as a predictor of subsequent remission in a trial of electroconvulsive therapy (ECT), although early improvement with ECT appeared to provide a higher PPV than NPV (24, 25, 26). Thus, the preponderance of evidence supports the importance of early improvement (or lack thereof) in predicting later remission and response in patients with major depressive disorder. Although we identified five factors (allocation to Aug‐ARI, more lifetime episodes of depression, less severe suicidal ideation, less anxiety, and a higher baseline quality of life score) that influenced achieving early improvement, the effect sizes of the influence of these factors were of a small magnitude (Cohen's d=0.12–0.25).

The present study bolsters the proposed use of the lack of early improvement as a predictor of failure to achieve remission with the current medication. In fact, in the VAST‐D study, the NPV for early improvement was over 92%. The lack of early improvement contributes to identifying a majority of those who will not ultimately demonstrate remission of symptoms with the current treatment, even if the dosage is increased to the optimal therapeutic dosage. Therefore, if there is not at least a 20% drop from the baseline QIDS‐C score by the end of week 2, there is <8% chance of achieving remission, just over a one‐in‐three (38%) chance of reaching the response criterion, and a five‐eighths (62%) chance of achieving greater than minimal improvement at the end of week 12 with continuation of the medication. In contrast to the prediction of remission, when predicting response and greater than minimal improvement, PPV is generally a better predictor than NPV (Figure 1). The predictive ability of PPV did not differ across treatment groups.

Those who did not achieve early improvement were nearly twice as likely to be withdrawn from the study than those who achieved early improvement (30% vs. 15%, respectively). Study withdrawal may account, at least in part, for the low remission and response rates among patients who did not experience early improvement. It would be important to learn whether more perseverance would have resulted in better outcomes for some of these patients. The present results suggest that a change in intervention is likely warranted relatively early in a medication trial if early improvement is not evident. However, specific patient groups may benefit from a longer duration of the intervention.

Identifying the characteristics of patients who would benefit from additional time is important, as is developing strategies to enhance treatment adherence when improvement is slower than anticipated. Evaluation of the factors influencing a false negative outcome sheds some light on this issue. Participants who did not show early improvement but achieved remission by the end of week 12 (false negative outcome) were more likely to have lower baseline QIDS‐C scores, fewer adverse childhood experiences, lower baseline Beck Anxiety Inventory score, lower C‐SSRS scores, and higher baseline quality of life (Q‐LES‐Q‐SF) scores. These findings are similar to the factors influencing inclusion in the early improvement group, but the effect sizes were much larger among the participants classified as having false negative outcomes (0.37–0.84 vs. 0.12–0.25, respectively).

The use of early clinical improvement to predict remission has been reviewed by Lam (27). Four basic points were supported in the review: most improvement occurs during the first 2 weeks of treatment (28), early improvement differentiates SSRIs from placebo (29), early improvement is likely to be sustained (30), and early improvement predicts later remission (31) and better psychosocial functioning (32). Our data are consistent with findings that most of the improvement occurs early and is sustained and that there is utility in the use of early improvement or lack thereof to predict remission. We cannot comment on comparisons with placebo, because we did not use such a control in the VAST‐D study. Although psychosocial functioning as an outcome measure is not addressed here, subsequent VAST‐D reports will evaluate the role of psychosocial functioning and quality of life in these patients.

Does the use of early improvement as a predictor of remission make a difference in clinical decisions? Only one study has tested a strategy of changing the clinical management when early improvement (in the first 2 weeks) was not achieved during an initial trial of the antidepressant escitalopram (33). Only 192 of 879 participants (22%) in the Tadić et al. study met the predetermined criteria to enter the comparison group of early (week 2) medication change (to venlafaxine) or continuation of treatment as usual (escitalopram). The chosen endpoint of that study was remission as measured by the Hamilton Depression Rating Scale at week 8. While the data showed only a nonsignificant trend in the direction of early medication change providing a better outcome, a major confounding issue in the Tadić et al. study was that more patients in the treatment‐as‐usual group ultimately received the alternative intervention, venlafaxine, than those who had been allocated to switch to venlafaxine. In contrast to that trial, the VAST‐D trial did not allow switching of treatments after initial assignment.

### Strengths and Limitations

One of the strengths of the present analysis of the VAST‐D data was the availability of a large patient population who received frequent, closely monitored visits with dosaging guided by measurement‐based care. A second strength is that the study population focused on patients who had inadequate response to prior treatment for depression. These factors suggest that this study was ideally suited to determine the predictive value of early improvement. Because of the large patient population and multiple assessment visits early in the trial, we were able to bolster evidence provided by existing studies on the utility of effectively using early improvement (or lack thereof) as a guide for clinical management. Comparing our findings in a large sample of patients inadequately responding to an initial antidepressant trial with prior studies addressing the role of early improvement in initial antidepressant trials, it is apparent that the utility of determining the presence of early improvement is robust across clinical populations.

This study has some limitations. It is possible that some component of early improvement may be associated with the expectation of benefit associated with entering a randomized trial. Despite this concern, treatment duration in the trial had no impact on PPV. In contrast, there were modest changes in the NPV over time, which were greatest at the end of week 6 (Figure 1), consistent with an earlier report (10). Although the VAST‐D study was conducted in a diverse sample with regard to most baseline characteristics (6, 13), the patient population was predominantly male (approximately 85%), which may cause some generalizability issues in populations with a greater proportion of women. Also, on average, participants were below the target dosage for their augmenting agents or bupropion when early improvement was assessed. While full characterization of factors influencing remission may require taking into account the optimal dosages of antidepressant medications, it is encouraging that in the present study we could use the absence of early improvement to predict likely failure to achieve remission before the full antidepressant dosage was achieved. However, the ultimate value of early improvement depends on whether changing interventions at the end of week 2 produces better outcomes.

Results must also be interpreted in the context of VAST‐D being a “next‐step” treatment study of patients who had already experienced inadequate response to at least one antidepressant trial. Thus, overall remission rates were relatively low, ranging from 22% for patients in the Switch‐BUP group to 29% for those in the Aug‐ARI group. These low remission rates resulted in a lower ceiling for the PPV. Higher overall remission rates were achieved in the initial treatment phase of the Sequenced Treatment Alternatives to Relieve Depression (STAR*D) study with “first‐step” trials (6) and these likely would have been associated with higher PPVs. These caveats aside, a majority of patients seen in clinical settings ultimately require next‐step strategies, and the results of this study are directly applicable to this large and important patient group.

### Importance of Findings

Through this analysis, we reinforced existing literature that supports the utility of using early improvement in patients taking antidepressant medication to predict later remission, response, and greater than minimal improvement. Also, we were able to identify an optimal time for assessing early improvement. The predictive importance of lack of early improvement is based on the assumption that standard assessments of depression severity are obtained at least at baseline and at the end of week 2 of each new medication intervention.

### Future Research

The recognition that lack of early improvement following initiation of an antidepressant medication regimen tells us only that the current therapy—even allowing for dosage escalation—is unlikely to be effective. However, the lack of early improvement does not tell us what the next step should be. The utility of using the absence of early improvement to enhance clinical outcomes should be evaluated in randomized controlled trials that test whether continuing the current treatment for a longer duration or switching to an alternative intervention is more effective for those failing to show early improvement.

## Supporting information

Supplementary Material

## Footnotes

a QIDS‐C, Quick Inventory of Depressive Symptomatology–Clinician Rated. Because of withdrawals, the number of participants included in the analysis for remission was 1,458; 1,426; 1,367; and 1,283 for weeks 1, 2, 4, and 6, respectively, and the number of participants included in the analysis for response and minimal improvement was 1,108; 1,112; 1,114; and 1,108 for weeks 1, 2, 4, and 6, respectively.

a Aug‐ARI, augmentation with aripiprazole; Aug‐BUP, augmentation with bupropion‐SR; Switch‐Bup, switching to another antidepressant (i.e., bupropion‐SR). Increasing sensitivity and specificity are plotted for 10%, 20%, 30%, 40%, and 50% reductions from baseline Quick Inventory of Depressive Symptomatology–Clinician Rated score at week 2, respectively, for the three medication regimens. Area‐under‐the‐curve comparisons by chi‐square analysis did not support an influence of treatment assignment on prediction of remission by early improvement (Switch‐BUP vs. Aug‐BUP, χ^2^=0.056, p=0.81; Switch‐BUP vs. Aug‐ARI, χ^2^=0.0003, p=0.99; Aug‐BUP vs. Aug‐ARI, χ^2^=0.054, p=0.82).
