# Supplementary material for: Predictability of Nonremitting Depression After First 2 Weeks of Antidepressant Treatment: A VAST‐D Trial Report
Source: Psychiatr Res Clin Pract. 2019 Oct 3;1(2):58–67. doi: 10.1176/appi.prcp.20190003 (PMC9176018; doi:10.1176/appi.prcp.20190003)
Supplement: Supplementary file 1 — Supplementary Material [file RCP2-1-58-s001.pdf]

Supplement A

Location of Work: CSP #576 was performed across 35 Veterans Affairs (VA) medical centers across the U.S., including: Atlanta VA Medical Center, Atlanta, Georgia; Bruce W. Carter VA Medical Center, Miami, Florida; Central Texas Veterans Healthcare System, Temple, Texas; Charles George VA Medical Center, Asheville, North Carolina; Cincinnati VA Medical Center, Cincinnati, Ohio; Clement J. Zablocki VA Medical Center, Milwaukee, Wisconsin; Edward Hines Jr VA Hospital, Hines, Illinois; James A Haley VA Hospital, Tampa, Florida; Kansas City VA Medical Center, Kansas City, Missouri; Long Beach VA Healthcare System, Long Beach, California; Louis A. Johnson VA Medical Center, Clarksburg, West Virginia; Louis Stokes VA Medical Center and Case Western Reserve University School of Medicine, Cleveland, Ohio; Memphis VA Medical Center, Memphis, Tennessee; Minneapolis VA Health Care System, Minneapolis, Minnesota; New Mexico VA Healthcare System, Albuquerque; Philadelphia VA Medical Center, Philadelphia, Pennsylvania; Phoenix VA Health Care System, Phoenix, Arizona; Richard L Roudebush VA Medical Center, Indianapolis, Indiana; Salem VA Medical Center, Salem, Virginia; San Francisco VA Health Care System, San Francisco, California; Southern Arizona VA Healthcare System, Tucson; Tuscaloosa VA Medical Center, Tuscaloosa, Alabama; University of California, San Diego; VA Eastern Colorado Healthcare System, Denver; VA Loma Linda Healthcare System, Loma Linda, California; VA Maryland Healthcare System, Baltimore; VA Nebraska Western Iowa Healthcare System, Omaha; VA New England Mental Illness Research, Education, and Clinical Center, VA Connecticut Healthcare System, West Haven; VA Palo Alto Healthcare System, Palo Alto, California; VA Pittsburgh Healthcare System, Pittsburgh, Pennsylvania; VA Puget Sound Health Care System, American Lake/Tacoma,

Washington; VA San Diego Healthcare System, San Diego, California; Washington DC VA Medical Center; W.G. Hefner VA Medical Center, Salisbury, North Carolina; William S. Middleton Veterans Hospital, Madison, Wisconsin.

Supplement B

Average Dosages (mgs/day) of Bupropion or Aripiprazole for Each Treatment Group at Various Durations of the Treatment Intervention

Treatment Group	Drug	Baseline	Week 1	Week 2	Week 4	Week 6	Week 12
**Switch-BUP**	Bupropion	150	237	268	299	318	317
**Aug-BUP**	Bupropion	150	221	248	295	311	284
**Aug-ARI**	Aripiprazole	2	3	5	7	8	8

Average dose: the average dose prescribed at a follow-up visit, e.g., the average dose taken for the week prior to Week 2 assessment was approximately the Week 1 dose.
